# Successful treatment of eosinophilic esophagitis with upadacitinib prescribed for atopic dermatitis

**DOI:** 10.1002/jpr3.70170

**Published:** 2026-04-03

**Authors:** Nathalie Nguyen, Maureen Bauer

**Affiliations:** ^1^ Section of Pediatric Gastroenterology, Hepatology and Nutrition, Digestive Health Institute, Children's Hospital Colorado Aurora Colorado USA; ^2^ Department of Pediatrics Gastrointestinal Eosinophilic Diseases Program, University of Colorado School of Medicine Aurora Colorado USA; ^3^ Section of Pediatric Allergy & Immunology, Children's Hospital Colorado Aurora Colorado USA

**Keywords:** abrocitinib, JAK inhibitor, Janus kinase inhibitors, tofacitinib

## Abstract

We describe a pediatric patient treated with upadacitinib for atopic dermatitis (AD) who subsequently achieved sustained clinical and histologic remission of eosinophilic esophagitis (EoE). Upadacitinib is an oral small molecule selective Janus kinase 1 inhibitor that inhibits janus kinase‐signal transduction and activation of transcription (JAK‐STAT) pathway with wide inhibitory effects on the immune system, including Th2 signaling pathways. Therefore, it leads to broad suppression of inflammatory cytokines, including interleukin‐4 (IL‐4), interleukin‐13 (IL‐13), and thymic stromal lymphopoietin (TSLP), that play a central role in the pathogenesis of EoE. Given the broad signaling pathway, if a patient is started on upadacitinib for other indications, cessation of other therapies, particularly biologics like dupilumab, should be considered on a case‐by‐case basis.

## 1 INTRODUCTION

Eosinophilic esophagitis (EoE) is a chronic Th2 mediated allergic disease of the esophagus characterized by symptoms of esophageal dysfunction and histologically by esophageal eosinophilia.^1^ Historically, treatment of EoE includes proton pump inhibitors (PPI), swallowed topical corticosteroids (TCS) and diet elimination.^1^ Up to 90% of patients with EoE have other atopy and almost 50% of patients have concomitant atopic dermatitis (AD).^2^ In May 2022, the Food and Drug Administration (FDA) approved dupilumab as the first biologic for the treatment of EoE.^1^ Dupilumab is also approved for other conditions including AD, asthma, chronic rhinosinusitis with nasal polyps, bullous pemphigoid, prurigo nodularis, and chronic spontaneous urticaria. Therefore, in select patients with EoE and comorbid atopic conditions, dupilumab may be considered first line as it may be effective for multiple conditions.^1^ Upadacitinib is an oral small molecule selective Janus kinase 1 (JAK1) inhibitor approved in January 2022 for the treatment of moderate to severe AD in patients >12 years old.^3^, ^4^ Janus kinase‐signal transduction and activation of transcription (JAK‐STAT) signaling (particularly JAK1 inhibition) is downstream in activation of numerous Th2 cytokines including interleukin‐4 (IL‐4), interleukin‐13 (IL‐13), and thymic stromal lymphopoietin (TSLP) that are key drivers in AD and other atopic conditions.^5^ Herein we describe a patient who was treated with upadacitinib for AD which also effectively treated EoE.

## 2 CASE REPORT

A 15‐year‐old female with a history of severe AD, asthma, food allergies and EoE presented to gastroenterology clinic for further management. The patient was diagnosed with EoE at the age of 3 years where she presented with symptoms of feeding difficulties and vomiting (see Table 1). After diagnosis of EoE she was on different forms of TCS for several years which controlled her EoE including oral viscous budesonide and ciclesonide (see Table 1). The patient was evaluated by dermatology and was started on dupilumab 200 mg every 2 weeks (initially at dosing for AD). This was subsequently increased to 300 mg every week when dupilumab was approved for EoE, in line with recommended dosing for EoE. She underwent esophagogastroduodenoscopy with biopsy (EGD) on dupilumab that showed EoE was in histologic remission. Clinically, she had symptoms of nausea, abdominal pain, mild dysphagia with potatoes, and heartburn. Due to poorly controlled AD refractory to dupilumab, her dermatologist started her on upadacitinib 15 mg daily and stopped dupilumab. After 6 months, she underwent EGD on upadacitinib 15 mg daily as monotherapy where she remained in histological remission and had improvement in dysphagia. Repeat EGD done 1 year later demonstrated that she remained in clinical and histological remission on upadacitinib 15 mg daily (see Table 1). To date, she has not experienced any side effects or infections while on upadacitinib.

**Table 1:** Endoscopic and histologic findings with treatment of eosinophilic esophagitis.

Endoscopy	Treatment	EREFS	Histology
1	Index endoscopy‐ No treatment	Not available	Proximal: 30 eos/hpf Distal: 50 eos/hpf
2	Oral viscous budesonide 0.5 mg twice daily	Not available	Proximal: 0 eos/hpf Distal: 0 eos/hpf
3	Dupilumab 300 mg every week	0	Proximal: 0 eos/hpf Distal: 0 eos/hpf
4	Upadacitinib 15 mg daily	0	Proximal: 0 eos/hpf Distal: 0 eos/hpf
5	Upadacitinib 15 mg daily	0	Proximal: 0 eos/hpf Distal: 0 eos/hpf

Abbreviations: eos/hpf, eosinophils per high‐power field; EREFS, eosinophilic esophagitis endoscopic reference score.

## 3 DISCUSSION

Here, we describe a pediatric patient treated with upadacitinib for AD who subsequently achieved sustained clinical and histologic remission of EoE. Upadacitinib is FDA approved for the treatment of moderate to severe AD in adults and children >12 years old. Given its broad inhibitory effects, it is also FDA approved in adults for several inflammatory and autoimmune conditions including ulcerative colitis, Crohn's disease, rheumatoid arthritis, psoriatic arthritis, ankylosing spondylitis, and axial spondyloarthritis.^3^, ^4^ Upadacitinib and abrocitinib, both FDA approved for AD, are JAK1 inhibitors with greater inhibitory potency for JAK1 than for JAK2 or JAK3.^6^ First generation JAK inhibitors inhibit immune function more broadly, thus have a less favorable side effect profile. The second‐generation inhibitors, including upadacitinib and abrocitinib, have more selective inhibition, thus have a better safety profile.^5^ In trials, both upadacitinib and abrocitinib demonstrated >90% clinical improvement in most treated individuals with moderate to severe AD.^7^ In a randomized, blinded head‐to‐head trial of over 600 adults with moderate to severe AD, upadacitinib provided superior and more rapid skin clearance and itch relief compared to dupilumab.^8^ Thus, patients with severe AD who do not have improvement with dupilumab or other biologics may consider upadacitinib.^8^ This is perhaps not surprising, given JAK1 inhibition has broader immune inhibition compared to Dupilumab, targeting IL‐4 and IL‐13 (like Dupilumab) in addition to TSLP, Th22, and Th1 cytokines.^7^

One previous case report described a patient with refractory EoE who was effectively treated with Tofacitinib, a JAK1/JAK3 inhibitor.^9^ A case series recently described the use of upadacitinib for four patients with EoE refractory to dupilumab.^6^ Its effectiveness may be secondary to Th2 cytokines impacted by JAK1 inhibition which are also key drivers in EoE. While this patient remained in histologic remission with regard to EoE, we must highlight that the primary indication for upadacitinib was for AD. Upadacitinib can have inhibitory effects on both Th1 and Th2 signaling pathways and therefore has broad suppression of inflammatory cytokines.^4^ As the majority of patients with EoE have comorbid atopy, including AD, this is not a rare clinical situation. Therefore, if upadacitinib or abrocitinib is used for the treatment of AD, cessation of other therapies, particularly biologics like dupilumab, should be strongly considered. Additionally, one could also consider stopping other EoE therapies such as PPI or TCS.

The mechanism for treatment of EoE with upadacitinib is not fully understood but JAK inhibitors have broad inhibitory effects on both Th1 and Th2 pathways.^4^ We hypothesize that JAK inhibition of key Th1 and Th2 signaling pathways including IL‐4, IL‐13, and TSLP could effectively treat EoE, while also treating AD. In vitro studies showed that upadacitinib decreased eotaxin‐3 production, a known chemoattractant of eosinophils, in esophageal epithelial cells and esophageal smooth muscle cells.^10^ Given the broad inhibitory effects on these signaling pathways, upadacitinib and abrocitinib should be used with caution and the potential side effects should be considered. Currently, long‐term safety data in pediatrics is lacking and further data is needed. JAK inhibitors have a black boxed warning based on data from tofacitinib (pan‐JAK inhibition) in rheumatoid arthritis including include herpes zoster viral infections, thrombosis, malignancy, venous thromboembolism, death, serious infections and gastrointestinal perforation.^4^, ^7^ However, use of selective JAK inhibitors in AD is typically better tolerated with milder side effects such as upper respiratory tract infections, nausea, laboratory abnormalities such as thrombocytopenia, dyslipidemia, elevation of liver enzymes etc.^5^

## 4 CONCLUSION

In conclusion, JAK1 inhibitors, such as upadacitinib and abrocitinib, are approved for severe AD. They have inhibitory effects on both Th1 and Th2 signaling pathways with broad suppression of inflammatory cytokines. Given that the majority of patients with EoE have comorbid atopic disease, if upadacitinib is used for the treatment of AD, cessation of other therapies for EoE, particularly biologics like dupilumab, should be strongly considered. Further studies are needed to understand the mechanism, effectiveness, safety and role of upadacitinib in EoE.

## CONFLICT OF INTEREST STATEMENT

Nathalie Nguyen has served as a consultant for Regeneron, Sanofi and EvoEndo, Inc. Maureen Bauer has served as a consultant for Regeneron, Sanofi and receives royalties from Dynamed.

## ETHICS STATEMENT

Informed consent was obtained from the patient's parent for publication.

## References

[1] Dellon ES , Muir AB , Katzka DA , et al. ACG clinical guideline: diagnosis and management of eosinophilic esophagitis. Am J Gastroenterol. 2025;120:31‐59. 39745304 10.14309/ajg.0000000000003194

[2] Chehade M , Jones SM , Pesek RD , et al. Phenotypic characterization of eosinophilic esophagitis in a large multicenter patient population from the consortium for food allergy research. J Allerg Clin Immunol Pract. 2018;6:1534‐1544.e5. 10.1016/j.jaip.2018.05.038 PMC6132253 30075341

[3] Guttman‐Yassky E , Teixeira HD , Simpson EL , et al. Once‐daily upadacitinib versus placebo in adolescents and adults with moderate‐to‐severe atopic dermatitis (Measure Up 1 and Measure Up 2): results from two replicate double‐blind, randomised controlled phase 3 trials. Lancet. 2021;397:2151‐2168. 34023008 10.1016/S0140-6736(21)00588-2

[4] Mohamed MEF , Bhatnagar S , Parmentier JM , Nakasato P , Wung P . Upadacitinib: mechanism of action, clinical, and translational science. Clin Transl Sci. 2024;17:e13688. 37984057 10.1111/cts.13688 PMC10771099

[5] Butala S , Castelo‐Soccio L , Seshadri R , et al. Biologic versus small molecule therapy for treating moderate to severe atopic dermatitis: clinical considerations. J Allerg Clin Immunol Pract. 2023;11:1361‐1373. 10.1016/j.jaip.2023.03.011 PMC10164714 36948491

[6] Sia T , Bacchus L , Kumar A , et al. Upadacitinib for patients with eosinophilic esophagitis refractory to dupilumab. Allergol Int. 2025;1:010. 10.1016/j.alit.2025.11.010 41419361

[7] Guttman‐Yassky E , Renert‐Yuval Y , Brunner PM . Atopic dermatitis. Lancet. 2025;405:583‐596. 39955121 10.1016/S0140-6736(24)02519-4

[8] Blauvelt A , Teixeira HD , Simpson EL , et al. Efficacy and safety of upadacitinib vs dupilumab in adults with moderate‐to‐severe atopic dermatitis: a randomized clinical trial. JAMA Dermatol. 2021;157:1047‐1055. 34347860 10.1001/jamadermatol.2021.3023 PMC8340015

[9] Mendoza Alvarez LB , Liu X , Glover S . Treatment‐resistant eosinophilic oesophagitis successfully managed with tofacitinib. BMJ Case Rep. 2019;12:e232558. 10.1136/bcr-2019-232558 PMC6936404 31831517

[10] Nelson M , Zhang X , Podgaetz E , Melmed A , Spechler SJ , Souza RF . In human esophageal epithelial and muscle cells treated with Th2 cytokines, upadacitinib decreases eotaxin‐3 secretion and muscle tension. Gastroenterology. 2024;166:1166‐1169.e3 38364911 10.1053/j.gastro.2024.02.011

